# Knowledge, attitude and practices towards COVID-19 among Indian medical undergraduates: a questionnaire-based study

**DOI:** 10.12688/f1000research.131073.1

**Published:** 2023-04-03

**Authors:** Sharada Rai, Rakshatha Nayak, Anusha S Bhatt, Kudurugundi Basavaraju Vatsala, Chaithra Gowthuvalli Venkataramana, Animesh Jain

**Affiliations:** 1 Department of Pathology, Kasturba Medical College, Mangalore, Manipal Academy of Higher Education, Manipal, India; 2 Department of Community Medicine, Kasturba Medical College, Mangalore, Manipal Academy of Higher Education, Manipal, India

**Keywords:** Covid-19, Public Health, Prevention, Infectious disease, Pandemic

## Abstract

**Background**: The Coronavirus disease-19 (COVID-19) infection of severe acute respiratory syndrome coronavirus-2 (SARS-Cov-2) has emerged as a recent pandemic, increasing the need for epidemiological studies and studies on public health. Only some studies have evaluated the awareness of medical undergraduates in India and other countries, leading to a lack of literature.

**Methods**: This study is a descriptive cross-sectional questionnaire-based study conducted in Kasturba Medical College, Mangalore, India, between June to August 2020. An online survey using google forms was circulated among undergraduate medical students in India by a convenient sampling method for data collection. Descriptive analysis was derived based on frequencies and percentages, and the association with age, gender, and year of undergraduate training medical course was derived using the chi-square test.

**Results**: Altogether, 630 students from India responded to the survey, with a maximum response from students studying in the second year (38.7%). Nearly 63.85% of responders identified themselves as females. Knowledge regarding the human-to-human transmission of the virus, symptoms, complications, definition of “close contact, quarantine, and its indications was adequate among the students, with more than 70% correct responses. However, one-fourth of the students needed to gain more knowledge about masks. Respiratory hygiene was poor among 24.8%. Nearly 40% of students were unaware of the management of patients with COVID-19.

**Conclusion**: There is a need for regular quality training and institutional programs on infection control of COVID-19 and other infectious diseases across all Indian medical colleges to educate undergraduate medical students, who are future healthcare professionals, thus minimizing the risk of transmission and providing optimal care for patients.

## Introduction

The Coronavirus disease-19 (COVID-19), a recent pandemic due to severe acute respiratory syndrome coronavirus-2 (SARS-Cov-2) infection caused major health threats worldwide during the year 2020-2021. ^1^ The disease’s accelerated and extensive geographic spread during the pandemic posed significant challenges for administrative authorities and healthcare professionals.

All healthcare workers have a higher risk of infection due to patient exposure. Although undergraduate medical students are not actively involved in patient care, the possibility of them acquiring and transmitting the disease within healthcare centers and on medical college campuses is high. ^2^ Vaccinations like Covishield, Covaxin, Sputnik V, Corbevax, etc., are effectively used in India. Of these, Covishield is the most used, accounting for 81% of the total doses given. ^3^^–^^5^ It has drastically declined the number of new cases. ^6^ However, due to new variants like omicron, emerging frequently, and the Peltzman effect in the country, several cities are still facing on-and-off spikes in incidence and mortality rate, which has caused a shortage of healthcare facilities and front-line doctors. ^6^^,^^7^ Under the worst-case scenario, voluntary recruitment of medical undergraduates as a potential solution for lacking human resources may be beneficial. ^2^ The student’s and patient’s safety is of significant concern in such emergent circumstances. ^8^^,^^9^

Awareness about the disease, its transmission, risk factors, prevention, infection control, guidelines, and practices during patient care among healthcare workers and students is fundamental to preventing healthcare-associated transmission and infection. ^10^^–^^13^ Studies have been done to assess the understanding of COVID-19 among healthcare workers and the general population in various countries. ^14^^–^^22^ But there are only few studies that have been done evaluating the awareness of medical undergraduates in India, leading to a need for more literature. ^2^^,^^8^^,^^9^ The objective of the study is to determine the Knowledge, Attitudes, and Practices (KAP) of Indian medical undergraduate students regarding the COVID-19 pandemic, which can help us to recognize faulty practices and attitudes of the students towards COVID-19. In addition, it can help us plan and review our policies to implement best practices for controlling the COVID-19 disease.

## Methods

### Ethics

We obtained online written informed consent from all participants and approval from the Institutional Ethics Committee. (Kasturba Medical College, Mangalore, Reg. No. ECR/54/Inst/KA/2014/RR-17, Protocol No. IECKMCMLR-07/2020/218)

This study is a descriptive cross-sectional study. An online survey using Google Forms was circulated among 630 medical undergraduate students in India. With an assumption of 50% of the Indian Medical undergraduates having adequate awareness and 10% relative precision, for a 95% confidence interval, the sample size was calculated to be 384. Adding 20% non-response rate, the final sample size came to be 460. A self-administered questionnaire was developed which consisted of socio-demographic questions, 20 questions based on knowledge, 7 questions based on attitude, and 12 questions on practices related to COVID-19 disease, adapted from the current interim guidance and information for healthcare workers published by the World Health Organisation (WHO), updated on 12 ^th^ June 2020 ^23^^–^^33^ The authors approached their colleagues from other institutions throughout the country through whom the medical undergraduates were identified. Social media platforms like Facebook, WhatsApp, Twitter were also used to identify common groups containing medical undergraduates from the country. The survey was circulated through social media by creating a link to the questionnaire. Non-random (convenience and snowball) sampling was done. Undergraduates who were studying in India and willing to participate in the survey irrespective of their age, sex, gender or institution were included in the study. The gender and age of the students were considered based on the responses given by them in the online forms. The online responses were collected over a period of two months duration from 16th June to 15 ^th^ August 2020.

We analyzed the collected data using the Statistical Package for the Social Sciences, version 25. Only complete responses were considered for data analysis. Descriptive analysis using frequencies and percentages was done. At the same time, the chi-square test was done for association between KAP concerning age, gender, and year of undergraduate training medical course.

## Results

Six hundred thirty students from various medical colleges in India belonging to different states like Karnataka, Kerala, Tamil Nadu, Hyderabad, Gujarat, Rajasthan, Maharashtra, Madhya Pradesh, West Bengal, Goa etc. responded to the survey. Out of these, we accepted only 618 responses and rejected 12 due to incomplete or incorrect answers. Most students were in the second year of their undergraduate training course (38.7%), with the maximum response from students aged 20 years (31.7%). Nearly 63.85% of responders identified themselves as females, and 36.2% identified themselves as males. Most responses were seen from different institutes of Karnataka state, followed by Kerala and Gujarat. The primary source of information on COVID-19 among the students was through news media (78.6%), official international health organization sites (66.7%), social-media (63.6%), official government sites (62.6%), and journals (14.1%). The majority of the students had received formal training in hand hygiene over the last year (73.5%). Most responders (68.4%) knew that the virus causing COVID-19 was Severe Acute Respiratory Syndrome Coronavirus 2 (SARS-CoV-2), although only 50.5% of students knew it was initially called 2019-nCoV. Knowledge regarding the human-to-human transmission of the virus was adequate among the students, with 94.5% responses for droplet transmission, which is the most regular route of transmission, 53.6% responses for contact transmission, and 53.2% responses for airborne transmission. Only 7.9% knew that faeco-oral transmission is also rarely reported in COVID-19. The initial symptoms of COVID-19 were well-known among the students, with maximum responses for fever (88.2%) and sore throat (84.8%). Only 20.7% knew diarrhoea could also be a presenting symptom of COVID-19. Although most students (98.9%) were aware of respiratory complications of COVID-19, only 17.5% were aware of cardiac complications, and 12% were aware of renal complications. Among the respondents, 76.5% were able to define “close contact correctly.” The understanding of home quarantine (97.9%) and indications for quarantine (90.8%) were satisfactory, although the first-year students had lesser knowledge about the same (p<0.05). More than 96% of students were aware of personal protective equipment worn by health care professionals. The majority of students were aware that respirators (76.1%) and surgical masks (58.3%) were effective in preventing transmission in healthcare workers, and three-layered cotton masks are reasonable alternatives in a public setting (73.6%). However, 11.2% believed that single-layered mask and 7.4% of students thought that dust mask was helpful in prevention. 7.8% believed that surgical masks were washable and reusable. Although the majority were aware that respiratory and hand hygiene (99.2%) and personal protective equipment (96.4%) are most effective in preventing infection, few responses for measures like vaccination (28.8%), Prophylactic Chloroquine (26.7%), taking frequent hot water baths (24.3%) were also seen. Among the students 88.7% had knowledge about vulnerable groups who are at higher risk for infection. More than 90% of students were aware that those infected with COVID-19 might not develop severe symptoms, with female students being more aware than males (p<0.05). Final-year students were the most knowledgeable, and first-year students were the least aware (p<0.05). 88.5% knew that asymptomatic patients could transmit the disease, but first and second-year students were least knowledgeable about the same (p<0.05). Nearly 75% of students were unaware that the preferred disinfection method for visibly soiled hands is hand washing with soap rather than alcohol-based hand sanitizer, with final-year students being most aware and first-year students least aware. Only 68.8% knew there was a risk of transmission of the SARS-CoV-2 virus through food delivery/takeaway, with male students being more knowledgeable than females (p<0.05), and 61.7% knew that the risk of transmission from pets is very low. Still, nearly 48.9% of first-year students were unaware of the same (p<0.05). More than 95% of students knew there is currently no effective cure for COVID-19, but early symptomatic and basic treatment can help most patients recover from the infection.

Nearly 4.7% of students responded that they would hide their symptoms due to the stigma attached to COVID-19, especially females and those in the age group of 21-23 years. More than 94% of students were willing to seek medical attention if they develop symptoms, despite the stigma attached to COVID-19. Although female students responded that they are more likely to hide their symptoms, they are also more likely to seek medical attention than male students. Nearly 86% of students are aware that despite good immunity, they can get infected if they had contact with an infected patient. However, nearly 16.7% of students who belonged to the age group of 18-20 years were unaware of this. And those in the final year were more aware, which is statistically significant (p<0.05). More than 86% of students feel the urge to update themselves on the virus, especially female students, which is statistically significant (p value<0.05). Nearly 40% of students were unaware how to approach and treat patients with signs and symptoms of COVID-19. Female students and younger students have the least confidence. Students in the final year, especially those above 24 years of age, had maximum confidence, which was statistically significant (p-value <0.05). 94.8% of students were willing to take vaccination against COVID-19 if available. Those belonging to 2nd years and 4th years were more willing than others, which was statistically significant (p<0.05).

About 80.3% of students were willing to volunteer as healthcare providers to fight COVID-19. Even though the female students were less confident about the management, they were more eager to volunteer as health care providers compared to their male co-students. The majority of students avoided close contact like a handshake (96.6%), avoid meeting people with symptoms (83.8%) and going to crowded places during the pandemic (89.5%), and followed regular hand hygiene practices like washing with soap (96.8%) and alcohol-based hand sanitizers (86.9%). Female students were more cautious about handshakes and avoided going to crowded places compared to male students, which was statistically significant (p <0.05). Female students also tended to use alcohol-based sanitizers more than male students, which was statistically significant. (p<0.05). However, respiratory hygiene was not followed by 24.8%, of which the majority belonged to their final year and is statistically significant (p <0.05). Nearly 25.1% of students resided with vulnerable people such as elderly individuals and those with co-morbidities, most of whom were final-year students and above the age of 24, which is statistically significant (p <0.05). Nearly 49.7% consumed specific medicine/food to boost immunity, like multivitamins, proteins, herbal drinks, homemade remedies, and ayurvedic medications. Almost 99.4% of students wore a mask when they went outside during the pandemic.

## Discussion

The recent pandemic, COVID-19, caused by SARS-Cov-2, was first diagnosed in December 2019 in Wuhan, China. ^14^ People working in healthcare facilities but not in a clinical setting, like medical students and clerical staff, are also at risk of exposure. Their lack of knowledge can lead to person-person transmission and the spread of the disease. In India, medical colleges have conducted online classes and sessions for undergraduates due to many cases during the pandemic. After the third wave in 2022, the students were recruited back to the campus for regular on-site courses. ^34^ Despite vaccinations like Covishield, Covaxin, Sputnik V, Corbevax etc. effectively bringing down the overall incidence of cases in the country, some high-alert cities like Delhi and Mumbai experienced sudden surges in patients, especially after re-opening of schools and colleges with a significant mortality rate. ^3^^–^^7^ It is, therefore, essential to know the attitudes and practices of medical students regarding COVID-19 to avoid further transmission and avoid any fourth wave in the country.

Knowledge about the disease, route of transmission, prevention, identifying the high-risk groups, initial symptoms of illness, and early isolation of suspected individuals are the most critical steps toward disease control. ^35^^–^^37^

In our study, most students were aware of droplet transmission; however, less than half were aware of other common routes like airborne, contact, and faeco-oral transmission. ^14^^–^^21^

Most patients with COVID-19 experience a mild flu-like illness. They have a fever, cough, myalgia, and shortness of breath. Rarely some may experience atypical symptoms such as fatigue, reduced alertness, reduced mobility, diarrhoea, loss of appetite, delirium etc. without any febrile episodes. ^19^^,^^20^ Few patients are asymptomatic. Nearly 10-20% of patients have been reported to develop severe symptoms characterized by severe pneumonia, septic shock, acute respiratory distress syndrome, metabolic acidosis, multi-organ failure, and coagulation dysfunction demanding intensive care. ^19^^,^^20^ In our study, although many were aware of the initial respiratory symptoms and complications, few were knowledgeable about other symptoms like myalgia, diarrhoea, renal failure, and shock. Most students were also aware that not all patients develop the disease, and asymptomatic patients can transmit the disease.

Currently, no specific antiviral treatment is known to be effective in combating the disease. ^15^^,^^16^ However, nearly 20% of our responders believe that antibiotics and other myths, like hand dryers, hot water baths, etc., can prevent viral transmission.

The understanding of “close contact” and home quarantine and its indication was satisfactory among students. ^33^ There was adequate awareness about high-risk groups, such as those over 60, or with underlying comorbidities, smokers, pregnant women, malnourished and immune-compromised which was noteworthy as many resided with the vulnerable group during the pandemic. ^19, 38, 39^

Personal measures like hand hygiene, respiratory etiquette, masks and personal protective equipment, environmental cleaning, and disinfection at home effectively reduce transmission. ^20^ Though this was practiced by many, the standardized hand hygiene techniques for visibly soiled hands, which is handwashing with soap and water were not known by all. Moreover, respiratory hygiene methods, like using sanitary tissue while coughing or sneezing and its proper disposal, were practiced by only few. These faulty practices have to be addressed cautiously.

The knowledge about personal protective equipment was satisfactory. Almost all the students wore a mask when they go out. Most students were aware of the different masks available for protection, but nearly 20% believed that surgical masks were washable and reusable. Only half the responders knew respirators were the most effective mask for aerosol-generating procedures. ^40^ This could increase the transmission rate in health-care setups if not acknowledged.

Most students practiced social distancing during high-case alerts which was a good practice. Although most students in the study were willing to volunteer as healthcare providers to fight against COVID-19, only a few are aware and confident about proper treatment protocols for infected patients.

Currently, similar studies on medical students in India and other South Asian countries are limited. Studies show that the knowledge of undergraduate medical students in India about COVID-19 is unsatisfactory. ^2^^,^^8^^,^^9^ Similarly, this study suggests that despite satisfactory awareness, attitude, and practices among the majority of students, there is still scope for improvement. Moreover, in countries like India, where there is an acute shortage of front-line doctors, recruiting undergraduate medical students to serve in the voluntary front-line team against COVID-19 is a reasonable option. ^8^ Which further demands effective planning and education to enable better adherence to good practices, including pre-exposure and post-exposure prophylaxis, to prevent the spread COVID-19. Regular training courses in infection control, including theory and practical, can help medical teams face unexpected consequences.

The drawbacks of this study include the less-than-expected response from students and lack of proper representation from entire country, probably due to selection bias as all the institutions and states from the country couldn’t be approached due to lack of contact details. Using social media to share the questionnaire and online survey, also limited the study as not all students had social media accounts and well established internet is not widely available in remote areas of the country.

It has been three years since the first onset of COVID-19 in China. ^1^ But there is a lot of uncertainty concerning this virus’s key epidemiological, clinical, and virological characteristics, with several ongoing research on its virulence and spread. The cure or vaccination against this disease is still under trial and may require considerable time for its implementation and complete acceptance. ^4^^,^^41^ The misinformation provided by spurious organizational bodies and shared on social media has caused confusion and fear among the public. ^42^ Adequate knowledge about COVID-19 and updates on various guidelines among healthcare workers plays a crucial role in infection control and transmission prevention. Medical students can be considered an accessible and approachable source of information to their families and the public. They are essential in forming a link between the medical field and society during any time of health crisis. Necessary policies, educational programs, training, interactive sessions, and evaluation needs to be planned and modulated by administrative authorities, to avoid any unpreparedness in the face of similar emergencies in the future.

## Data Availability

Figshare: https://doi.org/10.6084/m9.figshare.22178414.v3. ^43^ This project contains the following underlying data:
• Data-Excel.xlsx Figshare: https://doi.org/10.6084/m9.figshare.22178423.v3. ^44^ This project contains the following underlying data:
• Statistical results.doc Figshare: https://doi.org/10.6084/m9.figshare.22180984.v2. ^45^ This project contains the following extended data:
• Questionnaire.docx Data are available under the terms of the Creative Commons Zero “No rights reserved” data waiver (CC0 1.0 Public domain dedication).

## References

[1] World Health Organization: *Coronavirus disease 2019 (COVID-19): Weekly Epidemiological Update 18 January 2022.* [Accessed 19 January 2022]. Reference Source

[2] Modi PD Nair G Uppe A : COVID-19 Awareness Among Healthcare Students and Professionals in Mumbai Metropolitan Region: A Questionnaire-Based Survey. *Cureus.* 2020;12(4):e7514. 10.7759/cureus.7514 32377462 PMC7198075

[3] World Health Organization: *The Bharat Biotech BBV152 COVAXIN vaccine against COVID-19: What you need to know.* [Accessed 11 February 2023]. Reference Source

[4] Vaccine information, ICMR New Delh: *COVID-19 vaccine.* [Accessed 19 February 2023]. Reference Source

[5] BBC News: *Covishield and Covaxin: What we know about India’s Covid-19 vaccines.* [Acessed 19 February 2023]. Reference Source

[6] Juyal D Pal S Thaledi S : COVID-19: The vaccination drive in India and the Peltzman effect. *J Fam Med Prim Care.* 2021;10(11):3945–3947. [Acessed 19 February 2023]. 10.4103/jfmpc.jfmpc_739_21 Reference Source 35136749 PMC8797072

[7] Deol T : *Fourth wave? COVID-19 cases rising in India as mask mandate lifted, schools reopen.* [Acessed 19 February 2023]. Reference Source

[8] Gupta L Agarwal V Davalbhakta S : A survey-based study on the knowledge, attitude, and the practices pertaining to the 2019 novel CoronaVirus infection amongst undergraduate medical students in India. *medRxiv.* 2020. [preprint]. [Accessed 16 June 2020]. 10.1101/2020.04.11.20061333

[9] Maheshwari S Gupta PK Sinha R : Knowledge, attitude, and practice towards coronavirus disease 2019 (COVID-19) among medical students: A cross-sectional study. *J Acute Dis.* 2020;9(3):100–104. [Accessed 16 June 2020]. 10.4103/2221-6189.283886

[10] World Health Organization: *Surveillance protocol for SARS-CoV-2 infection among health workers.* WHO;2020. [Accessed 16 June 2020]. Reference Source

[11] World Health Organization: *Assessment of risk factors for coronavirus disease 2019 (COVID-19) in health workers: protocol for a case-control study.* WHO;2020. [Accessed 16 June 2020]. Reference Source

[12] World Health Organization: *Coronavirus disease 2019 (COVID-19): outbreak: rights, roles and responsibilities of health workers, including key considerations for occupational safety and health.* Interim guidance;2020. [Accessed 16 June 2020]. Reference Source

[13] World Health Organization: *Origin of SARS-CoV-2.* WHO;2020. [Accessed 16 June 2020]. Reference Source

[14] Kamate SK Sharma S Thakar S : Assessing Knowledge, Attitudes and Practices of dental practitioners regarding the COVID-19 pandemic: A multinational study. *Dent Med Probl.* 2020;57(1):11–17. 10.17219/dmp/119743 32307930

[15] Umakanthan S Sahu P Ranade AV : Origin, Transmission, Diagnosis and Management of Coronavirus Disease 2019 (COVID). *Postgrad Med J Epub.* 2020;1–6. 10.1136/postgradmedj-2020-138234 PMC10016932 32563999

[16] Huynh G Nguyen TNH Tran VK : Knowledge and attitude toward COVID-19 among healthcare workers at District 2 Hospital, Ho Chi Minh City. *Asian Pac J Trop Med.* 2020;13:260. 10.4103/1995-7645.280396

[17] Alzoubi H Alnawaiseh N Al-Mnayyis A : COVID-19 - Knowledge, Attitude and Practice among Medical and Non-Medical University Students in Jordan. *J Pure Appl Microbiol.* 2020;14(1):17–24. 10.22207/JPAM.14.1.04

[18] Zhong BL Luo W Li HM : Knowledge, attitudes, and practices towards COVID-19 among Chinese residents during the rapid rise period of the COVID-19 outbreak: a quick online cross-sectional survey. *Int J Biol Sci.* 2020;16(10):1745–1752. 10.7150/ijbs.45221 32226294 PMC7098034

[19] Hezima A Aljafari A Aljafari A : Knowledge, attitudes, and practices of Sudanese residents towards COVID. *East Mediterr Health J.* 2020;26(6):646–651. 10.26719/emhj.20.076 32621498

[20] Rugarabamu S Ibrahim M Byanaku A : Knowledge, attitudes, and practices (KAP) towards COVID-19: A quick online cross-sectional survey among Tanzanian residents. *medRxiv.* 2020. [preprint]. 10.1101/2020.04.26.20080820

[21] Asraf H Garima T Singh BM : Knowledge, attitude and practice towards COVID 19 a survey from Nepal. *Asian J Med Sci.* 2020;11(3):6–11. 10.3126/ajms.v11i3.28485

[22] Acharya L Gundi M Ngo TD : *COVID-19-related knowledge, attitudes, and practices among adolescents and young people in Bihar and Uttar Pradesh, India.* New York: Population council;2020. [Accessed 16 June 2020]. Reference Source

[23] World Health Organization: *Contact tracing in the context of COVID-19.* Interim guidance;2020. [Accessed 12 June 2020]. Reference Source

[24] World Health Organization: *Infection prevention and control during health care when COVID-19 is suspected.* Interim guidance;2020. [Accessed 16 June 2020]. Reference Source

[25] World Health Organization: *Overview of public health and social measures in the context of COVID-19.* Interim guidance;2020. [Accessed 16 June 2020]. Reference Source

[26] World Health Organization: *Rational use of personal protective equipment for coronavirus disease (COVID-19) and considerations during severe shortages.* Interim Guidance;2020. [Accessed 12 June 2020]. Reference Source

[27] World Health Organization: *Advice on the use of masks in the context of covid-19.* Interim uidance;2020. [Accessed 18 June 2020]. Reference Source

[28] World Health Organization: *WHO Guidelines on Hand Hygiene in Health Care: a Summary.* WHO;2009. [Accessed 13 June 2020]. Reference Source

[29] World Health Organization: *Cleaning and disinfection of environmental surfaces in the context of COVID-19.* Interim guidance;2020. [Accessed 11 June 2020]. Reference Source

[30] World Health Organization: *Considerations for quarantine of individuals in the context of containment for coronavirus disease (covid 19).* Interim guidance;2020. [Accessed 13 June 2020]. Reference Source

[31] World Health Organization: *Algorithm for COVID-19 triage and referral COVID -19 Outbreak.* WHO regional office Manila;2020. [Accessed 11 June 2020]. Reference Source

[32] National Centre for Disease Control: *Guidelines for Setting up Isolation Facility/Ward.* Government of India;2020. [Accessed 12 June 2020]. Reference Source

[33] Government of India: *Guidelines for home quarantine.* 2020. [Accessed 12 June 2020]. Reference Source

[34] Jena PK : Impact of pandemic COVID-19 on education in India. *International Journal of Current Research.* 2020;12(07):12582–12586. Reference Source

[35] World Health Organisation: *Clinical management of COVID-19.* Interim guidance;2020. [Accessed 21 June 2020]. Reference Source

[36] Health Catalyst: Improving Population Health: Data-Driven Approach to Identifying and Engaging Patients with High Risk of Mortality from COVID-19. 2020. [Accessed 13 June 2020]. Reference Source

[37] Kar SK Verma N Saxena : Coronavirus Infection Among Children and Adolescents. Saxena S , editor. *Coronavirus Disease 2019 (COVID-19). Medical Virology: From Pathogenesis to Disease Control.* Singapore: Springer;2020. 10.1007/978-981-15-4814-7_7

[38] World Health Organization: *Multisystem inflammatory syndrome in children and adolescents with COVID-19.* Scientific Brief;2020. [Accessed 16 June 2020]. Reference Source

[39] World Health Organization: *Smoking and COVID-19.* Scientific Brief;2020. [Accessed 16 June 2020]. Reference Source

[40] Salvi SS : *In this pandemic and panic of COVID-19 what should doctors know about masks and respirators?* Pune: Pulmocare Research and Education (PURE) Foundation;2020. [Accessed 13 June 2020]. Reference Source

[41] Lahariya C : *Third wave of the COVID-19 pandemic in India: What lies ahead?* DownToEarth;2022. [Accessed 19 January 2022]. Reference Source

[42] Banarjee C : *India world’s biggest Covid misinformation source: Study.* The Times of India;2021. [Accessed 19 January 2022]. Reference Source

[43] Nayak R Rai S Jain A : Data table.Epub ahead of print 27 February 2023. 10.6084/m9.figshare.22178414.v3

[44] Nayak R Rai S Jain A : Statistical analysis.Epub ahead of print 27 February 2023. 10.6084/m9.figshare.22178423.v3

[45] Nayak R Rai S Padmanabha N : Questionnaire.Epub ahead of print 27 February 2023. 10.6084/m9.figshare.22180984.v2

