# Knowledge, attitude and practices towards COVID-19 among Indian medical undergraduates: a questionnaire-based study

**DOI:** 10.12688/f1000research.131073.2

**Published:** 2024-05-22

**Authors:** Sharada Rai, Rakshatha Nayak, Anusha S Bhatt, Kudurugundi Basavaraju Vatsala, Chaithra Gowthuvalli Venkataramana, Animesh Jain

**Affiliations:** 1 Department of Pathology, Kasturba Medical College, Mangalore, Manipal Academy of Higher Education, Manipal, Karnataka, 576104, India; 2 Department of Community Medicine, Kasturba Medical College, Mangalore, Manipal Academy of Higher Education, Manipal, Karnataka, 576104, India

**Keywords:** Covid-19, Public Health, Prevention, Infectious disease, Pandemic

## Abstract

**Background:**

The Coronavirus disease-19 (COVID-19) infection of severe acute respiratory syndrome coronavirus-2 (SARS-Cov-2) has emerged as a recent pandemic, increasing the need for epidemiological studies and studies on public health. Only some studies have evaluated the awareness of medical undergraduates in India and other countries, leading to a lack of literature.

**Methods:**

This study is a descriptive cross-sectional questionnaire-based study conducted in Kasturba Medical College, Mangalore, India, between June to August 2020. An online survey using Google Forms was circulated among undergraduate medical students in India by a convenient sampling method for data collection. Descriptive analysis was derived based on frequencies and percentages, and the association with age, gender, and year of undergraduate training medical course was derived using the chi-square test.

**Results:**

Altogether, 630 students from India responded to the survey, with a maximum response from students studying in the second year (38.7%). Nearly 63.85% of responders identified themselves as females. Knowledge regarding the human-to-human transmission of the virus, symptoms, complications, definition of “close contact, quarantine, and its indications was adequate among the students, with more than 70% correct responses. However, one-fourth of the students needed to gain more knowledge about masks. Respiratory hygiene was poor among 24.8%. Nearly 40% of students were unaware of the management of patients with COVID-19.

**Conclusion:**

There is a need for regular quality training and institutional programs on infection control of COVID-19 and other infectious diseases across all Indian medical colleges to educate undergraduate medical students, who are future healthcare professionals, thus minimizing the risk of transmission and providing standardized care for patients.

## Introduction

The Coronavirus disease-19 (COVID-19), a recent pandemic due to severe acute respiratory syndrome coronavirus-2 (SARS-Cov-2) infection caused major health threats worldwide during the year 2020-2021. ^1^ The disease’s accelerated and extensive geographic spread during the pandemic posed significant challenges for administrative authorities and healthcare professionals.

All healthcare workers have a higher risk of infection due to patient exposure. Although undergraduate medical students are not actively involved in patient care, the possibility of them acquiring and transmitting the disease within healthcare centers and on medical college campuses is high. ^2^ Vaccinations like Covishield, Covaxin, Sputnik V, Corbevax, etc., are effectively used in India. Of these, Covishield is the most used, accounting for 81% of the total doses given. ^3^^–^^5^ It has drastically declined the number of new cases. ^6^ However, due to new variants like omicron, emerging frequently, and the Peltzman effect in the country, several cities are still facing on-and-off spikes in incidence and mortality rate, which has caused a shortage of healthcare facilities and front-line doctors. ^6^^,^^7^ Under the worst-case scenario, voluntary recruitment of medical undergraduates as a potential solution for lacking human resources may be beneficial. ^2^ The student’s and patient’s safety is of significant concern in such emergent circumstances. ^8^^,^^9^

Awareness about the disease, its transmission, risk factors, prevention, infection control, guidelines, and practices during patient care among healthcare workers and students is fundamental to preventing healthcare-associated transmission and infection. ^10^^–^^13^ Studies have been done to assess the understanding of COVID-19 among healthcare workers and the general population in various countries. ^14^^–^^22^ But there are only few studies that have been done evaluating the awareness of medical undergraduates in India, leading to a need for more literature. ^2^^,^^8^^,^^9^ The objective of the study is to determine the Knowledge, Attitudes, and Practices (KAP) of Indian medical undergraduate students regarding the COVID-19 pandemic, which can help us to recognize faulty practices and attitudes of the students towards COVID-19. In addition, it can help us plan and review our policies to implement best practices for controlling the COVID-19 disease.

## Methods

### Ethics

We obtained online written informed consent from all participants and approval from the Institutional Ethics Committee. (Kasturba Medical College, Mangalore, Reg. No. ECR/54/Inst/KA/2014/RR-17, Protocol No. IECKMCMLR-07/2020/218)

This study is a descriptive cross-sectional study. An online survey using Google Forms was circulated among 630 medical undergraduate students in India. With an assumption of 50% of the Indian Medical undergraduates having adequate awareness and 10% relative precision, for a 95% confidence interval, the sample size was calculated to be 384. Adding 20% non-response rate, the final sample size came to be 460. A self-administered questionnaire was developed which consisted of socio-demographic questions, 20 questions based on knowledge, 7 questions based on attitude, and 12 questions on practices related to COVID-19 disease. ^45^ The questionnaire underwent content validation and was crafted by drawing from the latest interim guidance and information provided by the World Health Organization (WHO), as of June 12th, 2020. This process ensured that the questionnaire’s questions were in line with the standards and recommendations set forth by the WHO for healthcare workers during the pandemic. ^23^^–^^33^ The authors approached their colleagues from other institutions throughout the country through whom the medical undergraduates were identified. Social media platforms like Facebook, WhatsApp, Twitter were also used to identify common groups containing medical undergraduates from the country. The survey was circulated through social media by creating a link to the questionnaire. Non-random (convenience and snowball) sampling was done. Undergraduates who were studying in India and willing to participate in the survey irrespective of their age, sex, gender or institution were included in the study. The gender and age of the students were considered based on the responses given by them in the online forms. The online responses were collected over a period of two months duration from 16th June to 15 ^th^ August 2020.

We analyzed the collected data using the Statistical Package for the Social Sciences, version 25. Only complete responses were considered for data analysis. Descriptive analysis using frequencies and percentages was done. At the same time, the chi-square test was done for association between KAP concerning age, gender, and year of undergraduate training medical course.

## Results

Six hundred thirty students from various medical colleges in India belonging to different states like Karnataka, Kerala, Tamil Nadu, Hyderabad, Gujarat, Rajasthan, Maharashtra, Madhya Pradesh, West Bengal, Goa etc. responded to the survey. Out of these, we accepted only 618 responses and rejected 12 responses due to incomplete or incorrect answers. Most students were in the second year of their undergraduate training course (38.7%), with the maximum response from students aged 20 years (31.7%). Nearly 63.85% of responders identified themselves as females, and 36.2% identified themselves as males. Most responses were seen from different institutes of Karnataka state, followed by Kerala and Gujarat.

The study surveyed students’ knowledge, attitudes, and practices related to COVID-19 ( Table 1). Findings showed that news media was the primary information source for 78.6% of students, with 73.5% receiving hand hygiene training in the past year. Knowledge about COVID-19 varied, with 68.4% correctly identifying the virus as SARS-CoV-2. While awareness of transmission routes was generally high, gaps existed, such as recognizing faeco-oral transmission (7.9%). Symptoms like fever (88.2%) were well-known, but less awareness of diarrhea (20.7%). Understanding of complications varied, with fewer students aware of cardiac (17.5%) and renal complications (12%). Overall, knowledge improved with academic progression and was statistically significant (p<0.05). Attitudes towards seeking medical care were positive, despite stigma concerns. Most students (94.8%) expressed willingness to take a COVID-19 vaccine if available. Additionally, many students (80.3%) were willing to volunteer as healthcare providers with confidence level increasing with academic progression (p<0.05). Practices like hand hygiene were generally followed, although some lapses existed, particularly in respiratory hygiene. Demographic differences were observed, with female students typically exhibiting more cautious behaviors and greater awareness (p<0.05).

**Table 1.** Table showing the percentage of student responses for various categories under knowledge, attitude, and practices.

Category	Result
1. **Knowledge**	
The primary source of information on COVID-19 among students	
− News media	78.6%
− Official international health organization sites	66.7%
− Social-media	63.6%
− Official government sites	62.6%
− Journals	14.1%
Received formal training and knowledge of hand hygiene over the last year	73.5%
Knowledge of Virus causing COVID-19 (SARS-CoV-2)	68.4%
Knowledge about the virus being initially called as 2019-nCoV	50.5%
Knowledge of human-to-human transmission routes	
− Droplet transmission	94.5%
− Contact transmission	53.6%
− Airborne transmission	53.2%
− Faeco-oral transmission	7.9%
Knowledge of Initial symptoms of COVID-19	
− Fever	88.2%
− Sore throat	84.8%
− Diarrhoea	20.7%
Awareness of respiratory complications of COVID-19	98.9%
Awareness of cardiac complications	17.5%
Awareness of renal complications	12%
Understanding of “close contact”	76.5%
Knowledge of home quarantine	97.9%
Knowledge of indications for quarantine	90.8%
Awareness of personal protective equipment	>96%
Knowledge about effective preventive measures	
− Respirators	76.1%
− Surgical masks	58.3%
− Three-layered cotton masks	73.6%
Misconceptions about preventive measures	
− Single-layered mask being effective	11.2%
− Dust mask being effective	7.4%
− Surgical masks washable/reusable	7.8%
Awareness of the most effective preventive measure	99.2%
Awareness of vulnerable groups at higher risk	88.7%
Awareness of asymptomatic transmission	88.5%
Awareness of preferred disinfection method for soiled hands	25%
Awareness of transmission risks from food delivery	68.8%
Awareness of transmission risks from pets is low	61.7%
Awareness of the lack of an effective cure for COVID-19	95%
Willingness to hide symptoms due to stigma	4.7%
Willingness to seek medical attention despite stigma	94%
2. **Attitude**	
Seek medical attention despite stigma	94%
Urge to update knowledge on COVID-19	86%
Fear of potential infection despite good immunity	86%
Not Hide their symptoms due to stigma	95.3 %
Confidence in approach and treatment	60%
Willingness to take COVID-19 vaccination	94.8%
Willingness to volunteer as a healthcare provider	80.3%
3. **Practice**	
Adherence to preventive measures	
− Avoiding handshakes	96.6%
− Avoiding contact with symptomatic individuals	83.8%
− Avoiding crowded places	89.5%
− Hand hygiene practices (washing with soap)	96.8%
− Alcohol-based hand sanitizers	86.9%
Adherence to respiratory hygiene	76.2%
Students residing with vulnerable individuals	25.1%
Consumption of immunity-boosting medicines/foods	49.7%
Mask-wearing when going outside during the pandemic	99.4%

## Discussion

The emergence of COVID-19, originating in Wuhan, China, posed significant challenges globally in December 2019. ^14^ Beyond frontline healthcare workers, individuals in healthcare facilities outside clinical settings, including medical students and clerical staff, face exposure risks and potential sources of further infection. Amid India’s pandemic response, medical colleges transitioned to online classes, reverting to on-site courses after the third wave in 2022. After the third wave in 2022, the students were recruited back to the campus for regular on-site courses. ^34^ Despite vaccinations like Covishield, Covaxin, Sputnik V, Corbevax etc. effectively bringing down the overall incidence of cases in the country, some high-alert cities like Delhi and Mumbai experienced sudden surges in patients, especially after re-opening of schools and colleges with a significant mortality rate. ^3^^–^^7^ Therefore, a deeper understanding of medical students’ attitudes and practices is necessary to prevent further transmission and potential subsequent waves. Comprehensive knowledge of COVID-19, including its transmission routes, prevention strategies, identification of high-risk groups, symptomatology, and early isolation of infected individuals is pivotal for effective disease control. ^35^^–^^37^

While students demonstrated awareness of respiratory transmission, other routes like airborne, contact, and faeco-oral transmission were less recognized. ^14^^–^^21^ Although respiratory symptoms were well-known, awareness of atypical manifestations like fatigue, reduced alertness, reduced mobility, diarrhoea, loss of appetite, delirium etc. and complications like, renal failure, shock etc. varied. ^19^^,^^20^ Misconceptions persisted regarding preventive measures, with a notable proportion endorsing ineffective strategies like antibiotics and other myths, like hand dryers, hot water baths, etc. Currently, no specific antiviral treatment is known to be effective in combating the disease. ^15^^,^^16^ The understanding of “close contact” and home quarantine and its indication was satisfactory among students. ^33^ There was adequate awareness about high-risk groups, such as those over 60, or with underlying comorbidities, smokers, pregnant women, malnourished and immune-compromised which was noteworthy as many resided with the vulnerable group during the pandemic. ^19, 38, 39^

The study underscored significant gaps in understanding standardized hand hygiene and respiratory etiquette, critical in curbing transmission. While measures like hand hygiene, mask-wearing, and social distancing were are effective in reducing transmission, ^20^ and widely practiced by the students, there were glaring misconceptions. For instance, many weren’t familiar with proper handwashing techniques for visibly soiled hands or the importance of respiratory hygiene, like using tissues when coughing or sneezing, and their proper disposal. Addressing these shortcomings requires careful attention. Moreover, there were misconceptions about mask types and reuse, potentially risking transmission in healthcare settings. Only half the responders knew respirators were the most effective mask for aerosol-generating procedures. ^40^ Despite commendable practices like social distancing during high-case alerts and a willingness among students to volunteer in healthcare, awareness of proper treatment protocols for COVID-19 patients was lacking.

Similar studies in other countries, especially among South Asian countries and India, reveal inadequate COVID-19 knowledge among medical students. ^2, 8, 9^^,^^16^^–^^22^ Similarly, this study suggests that despite satisfactory awareness, attitude, and practices among the students, there is still scope for improvement. Moreover, in countries like India, where there is an acute shortage of front-line doctors, recruiting undergraduate medical students to serve in the voluntary front-line team against COVID-19 is a reasonable option. ^8^ Which further demands effective planning and education to enable better adherence to good practices, including pre-exposure and post-exposure prophylaxis, to prevent the spread of COVID-19. Regular training in infection control, including theory and practical aspects, is imperative to prepare medical teams for unforeseen challenges.

The drawbacks of this study include the less-than-expected response from students and lack of proper representation from the entire country, probably due to selection bias as all the institutions and states couldn’t be approached due to the lack of contact details. Using social media to share the questionnaire and online survey, also limited the study as not all students had social media accounts and well-established internet is not widely available in remote areas of the country.

## Conclusion

Three years since the initial outbreak in China, COVID-19’s key characteristics remain uncertain, prompting ongoing research. Vaccines and cures are still in trial phases, with implementation and acceptance requiring time. ^4^^,^^41^ Misinformation provided by spurious organizational bodies and shared on social media amplifies public anxiety, ^42^ highlighting the vital role of healthcare workers in disseminating accurate information for infection control and prevention. Adequate knowledge about COVID-19 and updates on various guidelines among healthcare workers play a crucial role in infection control and transmission prevention. Medical students, as accessible sources of information, can bridge the gap between the medical community and society during health crises. But this demands comprehensive policies, educational initiatives, and interactive sessions, orchestrated by administrative authorities, to ensure preparedness for future emergencies.

## Data Availability

Figshare: https://doi.org/10.6084/m9.figshare.22178414.v3. ^43^ This project contains the following underlying data:
• Data-Excel.xlsx Figshare: https://doi.org/10.6084/m9.figshare.22178423.v3. ^44^ This project contains the following underlying data:
• Statistical results.doc Figshare: https://doi.org/10.6084/m9.figshare.22180984.v2. ^45^ This project contains the following extended data:
• Questionnaire.docx Data are available under the terms of the Creative Commons Zero “No rights reserved” data waiver (CC0 1.0 Public domain dedication).

## References

[1] World Health Organization: *Coronavirus disease 2019 (COVID-19): Weekly Epidemiological Update 18 January 2022.* [Accessed 19 January 2022]. Reference Source

[2] Modi PD Nair G Uppe A : COVID-19 Awareness Among Healthcare Students and Professionals in Mumbai Metropolitan Region: A Questionnaire-Based Survey. *Cureus.* 2020;12(4):e7514. 10.7759/cureus.7514 32377462 PMC7198075

[3] World Health Organization: *The Bharat Biotech BBV152 COVAXIN vaccine against COVID-19: What you need to know.* [Accessed 11 February 2023]. Reference Source

[4] Vaccine information, ICMR New Delh: *COVID-19 vaccine.* [Accessed 19 February 2023]. Reference Source

[5] BBC News: *Covishield and Covaxin: What we know about India’s Covid-19 vaccines.* [Acessed 19 February 2023]. Reference Source

[6] Juyal D Pal S Thaledi S : COVID-19: The vaccination drive in India and the Peltzman effect. *J Fam Med Prim Care.* 2021;10(11):3945–3947. [Acessed 19 February 2023]. 10.4103/jfmpc.jfmpc_739_21 Reference Source 35136749 PMC8797072

[7] Deol T : *Fourth wave? COVID-19 cases rising in India as mask mandate lifted, schools reopen.* [Acessed 19 February 2023]. Reference Source

[8] Gupta L Agarwal V Davalbhakta S : A survey-based study on the knowledge, attitude, and the practices pertaining to the 2019 novel CoronaVirus infection amongst undergraduate medical students in India. *medRxiv.* 2020. [preprint]. [Accessed 16 June 2020]. 10.1101/2020.04.11.20061333

[9] Maheshwari S Gupta PK Sinha R : Knowledge, attitude, and practice towards coronavirus disease 2019 (COVID-19) among medical students: A cross-sectional study. *J Acute Dis.* 2020;9(3):100–104. [Accessed 16 June 2020]. 10.4103/2221-6189.283886

[10] World Health Organization: *Surveillance protocol for SARS-CoV-2 infection among health workers.* WHO;2020. [Accessed 16 June 2020]. Reference Source

[11] World Health Organization: *Assessment of risk factors for coronavirus disease 2019 (COVID-19) in health workers: protocol for a case-control study.* WHO;2020. [Accessed 16 June 2020]. Reference Source

[12] World Health Organization: *Coronavirus disease 2019 (COVID-19): outbreak: rights, roles and responsibilities of health workers, including key considerations for occupational safety and health.* Interim guidance;2020. [Accessed 16 June 2020]. Reference Source

[13] World Health Organization: *Origin of SARS-CoV-2.* WHO;2020. [Accessed 16 June 2020]. Reference Source

[14] Kamate SK Sharma S Thakar S : Assessing Knowledge, Attitudes and Practices of dental practitioners regarding the COVID-19 pandemic: A multinational study. *Dent Med Probl.* 2020;57(1):11–17. 10.17219/dmp/119743 32307930

[15] Umakanthan S Sahu P Ranade AV : Origin, Transmission, Diagnosis and Management of Coronavirus Disease 2019 (COVID). *Postgrad Med J Epub.* 2020;1–6. 10.1136/postgradmedj-2020-138234 PMC10016932 32563999

[16] Huynh G Nguyen TNH Tran VK : Knowledge and attitude toward COVID-19 among healthcare workers at District 2 Hospital, Ho Chi Minh City. *Asian Pac J Trop Med.* 2020;13:260. 10.4103/1995-7645.280396

[17] Alzoubi H Alnawaiseh N Al-Mnayyis A : COVID-19 - Knowledge, Attitude and Practice among Medical and Non-Medical University Students in Jordan. *J Pure Appl Microbiol.* 2020;14(1):17–24. 10.22207/JPAM.14.1.04

[18] Zhong BL Luo W Li HM : Knowledge, attitudes, and practices towards COVID-19 among Chinese residents during the rapid rise period of the COVID-19 outbreak: a quick online cross-sectional survey. *Int J Biol Sci.* 2020;16(10):1745–1752. 10.7150/ijbs.45221 32226294 PMC7098034

[19] Hezima A Aljafari A Aljafari A : Knowledge, attitudes, and practices of Sudanese residents towards COVID. *East Mediterr Health J.* 2020;26(6):646–651. 10.26719/emhj.20.076 32621498

[20] Rugarabamu S Ibrahim M Byanaku A : Knowledge, attitudes, and practices (KAP) towards COVID-19: A quick online cross-sectional survey among Tanzanian residents. *medRxiv.* 2020. [preprint]. 10.1101/2020.04.26.20080820

[21] Asraf H Garima T Singh BM : Knowledge, attitude and practice towards COVID 19 a survey from Nepal. *Asian J Med Sci.* 2020;11(3):6–11. 10.3126/ajms.v11i3.28485

[22] Acharya L Gundi M Ngo TD : *COVID-19-related knowledge, attitudes, and practices among adolescents and young people in Bihar and Uttar Pradesh, India.* New York: Population council;2020. [Accessed 16 June 2020]. Reference Source

[23] World Health Organization: *Contact tracing in the context of COVID-19.* Interim guidance;2020. [Accessed 12 June 2020]. Reference Source

[24] World Health Organization: *Infection prevention and control during health care when COVID-19 is suspected.* Interim guidance;2020. [Accessed 16 June 2020]. Reference Source

[25] World Health Organization: *Overview of public health and social measures in the context of COVID-19.* Interim guidance;2020. [Accessed 16 June 2020]. Reference Source

[26] World Health Organization: *Rational use of personal protective equipment for coronavirus disease (COVID-19) and considerations during severe shortages.* Interim Guidance;2020. [Accessed 12 June 2020]. Reference Source

[27] World Health Organization: *Advice on the use of masks in the context of covid-19.* Interim uidance;2020. [Accessed 18 June 2020]. Reference Source

[28] World Health Organization: *WHO Guidelines on Hand Hygiene in Health Care: a Summary.* WHO;2009. [Accessed 13 June 2020]. Reference Source

[29] World Health Organization: *Cleaning and disinfection of environmental surfaces in the context of COVID-19.* Interim guidance;2020. [Accessed 11 June 2020]. Reference Source

[30] World Health Organization: *Considerations for quarantine of individuals in the context of containment for coronavirus disease (covid 19).* Interim guidance;2020. [Accessed 13 June 2020]. Reference Source

[31] World Health Organization: *Algorithm for COVID-19 triage and referral COVID -19 Outbreak.* WHO regional office Manila;2020. [Accessed 11 June 2020]. Reference Source

[32] National Centre for Disease Control: *Guidelines for Setting up Isolation Facility/Ward.* Government of India;2020. [Accessed 12 June 2020]. Reference Source

[33] Government of India: *Guidelines for home quarantine.* 2020. [Accessed 12 June 2020]. Reference Source

[34] Jena PK : Impact of pandemic COVID-19 on education in India. *International Journal of Current Research.* 2020;12(07):12582–12586. Reference Source

[35] World Health Organisation: *Clinical management of COVID-19.* Interim guidance;2020. [Accessed 21 June 2020]. Reference Source

[36] Health Catalyst: Improving Population Health: Data-Driven Approach to Identifying and Engaging Patients with High Risk of Mortality from COVID-19. 2020. [Accessed 13 June 2020]. Reference Source

[37] Kar SK Verma N Saxena : Coronavirus Infection Among Children and Adolescents. Saxena S , editor. *Coronavirus Disease 2019 (COVID-19). Medical Virology: From Pathogenesis to Disease Control.* Singapore: Springer;2020. 10.1007/978-981-15-4814-7_7

[38] World Health Organization: *Multisystem inflammatory syndrome in children and adolescents with COVID-19.* Scientific Brief;2020. [Accessed 16 June 2020]. Reference Source

[39] World Health Organization: *Smoking and COVID-19.* Scientific Brief;2020. [Accessed 16 June 2020]. Reference Source

[40] Salvi SS : *In this pandemic and panic of COVID-19 what should doctors know about masks and respirators?* Pune: Pulmocare Research and Education (PURE) Foundation;2020. [Accessed 13 June 2020]. Reference Source

[41] Lahariya C : *Third wave of the COVID-19 pandemic in India: What lies ahead?* DownToEarth;2022. [Accessed 19 January 2022]. Reference Source

[42] Banarjee C : *India world’s biggest Covid misinformation source: Study.* The Times of India;2021. [Accessed 19 January 2022]. Reference Source

[43] Nayak R Rai S Jain A : Data table.Epub ahead of print 27 February 2023. 10.6084/m9.figshare.22178414.v3

[44] Nayak R Rai S Jain A : Statistical analysis.Epub ahead of print 27 February 2023. 10.6084/m9.figshare.22178423.v3

[45] Nayak R Rai S Padmanabha N : Questionnaire.Epub ahead of print 27 February 2023. 10.6084/m9.figshare.22180984.v2

