# Supplementary material for: Knowledge, attitude and practices towards COVID-19 among Indian medical undergraduates: a questionnaire-based study
Source: F1000Res. 2024 May 22;12:361. Originally published 2023 Apr 3. [Version 2] doi: 10.12688/f1000research.131073.2 (PMC11126899; doi:10.12688/f1000research.131073.2)
Supplement: Peer review history

# Reviewer report: Reviewer response for version 2

**DOI:** 10.5256/f1000research.166044.r281277

**Published:** 2024-05-24

**Authors:** Bodrun Naher Siddiquea

**Affiliations:** 1 Department of Epidemiology and Preventive Medicine, Monash University, Melbourne, Victoria, Australia

The authors have addressed my earlier comments to the best of their ability.

Is the work clearly and accurately presented and does it cite the current literature?

Partly

If applicable, is the statistical analysis and its interpretation appropriate?

Partly

Are all the source data underlying the results available to ensure full reproducibility?

Yes

Is the study design appropriate and is the work technically sound?

Partly

Are the conclusions drawn adequately supported by the results?

Partly

Are sufficient details of methods and analysis provided to allow replication by others?

Partly

Reviewer Expertise:

Both communicable and non-communicable diseases.

I confirm that I have read this submission and believe that I have an appropriate level of expertise to confirm that it is of an acceptable scientific standard.

# Reviewer report: Reviewer response for version 1

**DOI:** 10.5256/f1000research.143880.r227468

**Published:** 2024-05-15

**Authors:** Moawia Gameraddin

**Affiliations:** 1 Taibah University, Al-Madinah, Saudi Arabia

I did not find Tables and figures. The results section did not cite the tables and figures. I recommend revising the manuscript to provide the tables and figures, and the citation of them in the results section.

 A paper without tables and figures should be not be considered for indexing.

**Introduction: **suitable and expressive.

 The objectives are clear, and so is the presentation.

**Results:**

 This part is lacking the Tables and figures. I recommend the following:

1. The authors should add the tables and figures.
2. There is no citations for Tables and Figures in the results section.

**Decision: The paper needs major revision to add the tables and figures.**

Is the work clearly and accurately presented and does it cite the current literature?

Partly

If applicable, is the statistical analysis and its interpretation appropriate?

No

Are all the source data underlying the results available to ensure full reproducibility?

Partly

Is the study design appropriate and is the work technically sound?

Partly

Are the conclusions drawn adequately supported by the results?

Partly

Are sufficient details of methods and analysis provided to allow replication by others?

Partly

Reviewer Expertise:

1/Radiological sciences and medical imaging

I confirm that I have read this submission and believe that I have an appropriate level of expertise to state that I do not consider it to be of an acceptable scientific standard, for reasons outlined above.

# Reviewer report: Reviewer response for version 1

**DOI:** 10.5256/f1000research.143880.r168658

**Published:** 2024-04-29

**Authors:** Utsav Raj

**Affiliations:** 1 Department of Community and Family Medicine, All India Institute of Medical Sciences, Raipur, Chhattisgarh, India

Methods:

 Strengths: 

1. The methodology and statistical analysis are intricately detailed, bolstering the study's reliability.
2.  The inclusion of references for questionnaire preparation and data availability, elucidating question details and choices, enhances its credibility and ensures its value for future reference.

Results:

1. Weakness (minor): Presenting the results in paragraphs rather than in tabular format diminishes readability for the audience.

Discussion and Conclusion:

 Strengths: 

1. The discussion adeptly compares and analyses findings in relation to other studies, enriching its depth.
2. The conclusion underscores the imperative of comprehensive training for healthcare professionals worldwide to tackle unforeseen challenges effectively, advocating for the development of safer and better strategies.
3. The study holds relevance for a broad spectrum of reader's 

Is the work clearly and accurately presented and does it cite the current literature?

Yes

If applicable, is the statistical analysis and its interpretation appropriate?

Yes

Are all the source data underlying the results available to ensure full reproducibility?

Yes

Is the study design appropriate and is the work technically sound?

Yes

Are the conclusions drawn adequately supported by the results?

Yes

Are sufficient details of methods and analysis provided to allow replication by others?

Yes

Reviewer Expertise:

Public heath

I confirm that I have read this submission and believe that I have an appropriate level of expertise to confirm that it is of an acceptable scientific standard.

# Reviewer report: Reviewer response for version 1

**DOI:** 10.5256/f1000research.143880.r181439

**Published:** 2023-08-07

**Authors:** Bodrun Naher Siddiquea

**Affiliations:** 1 Department of Epidemiology and Preventive Medicine, Monash University, Melbourne, Victoria, Australia

**Methods**

1. Please mention about the validity of the questionnaire (tested or not) in the method section.
2. Please clearly mention about options of responses and scoring or of the questionnaire in the method section.

**Results**

1. Results presented in Tables and short summary will increase the readability of the article.

**Discussion and conclusion**

1. Please compare your results with published literature from other countries.
2. Too many paragraphs. 
3. Please write the conclusion under separate heading. 

Generally the paper needs proof reading to improve the grammatical errors.

Is the work clearly and accurately presented and does it cite the current literature?

Partly

If applicable, is the statistical analysis and its interpretation appropriate?

Partly

Are all the source data underlying the results available to ensure full reproducibility?

Yes

Is the study design appropriate and is the work technically sound?

Partly

Are the conclusions drawn adequately supported by the results?

Partly

Are sufficient details of methods and analysis provided to allow replication by others?

Partly

Reviewer Expertise:

Both communicable and non-communicable diseases.

I confirm that I have read this submission and believe that I have an appropriate level of expertise to confirm that it is of an acceptable scientific standard, however I have significant reservations, as outlined above.
